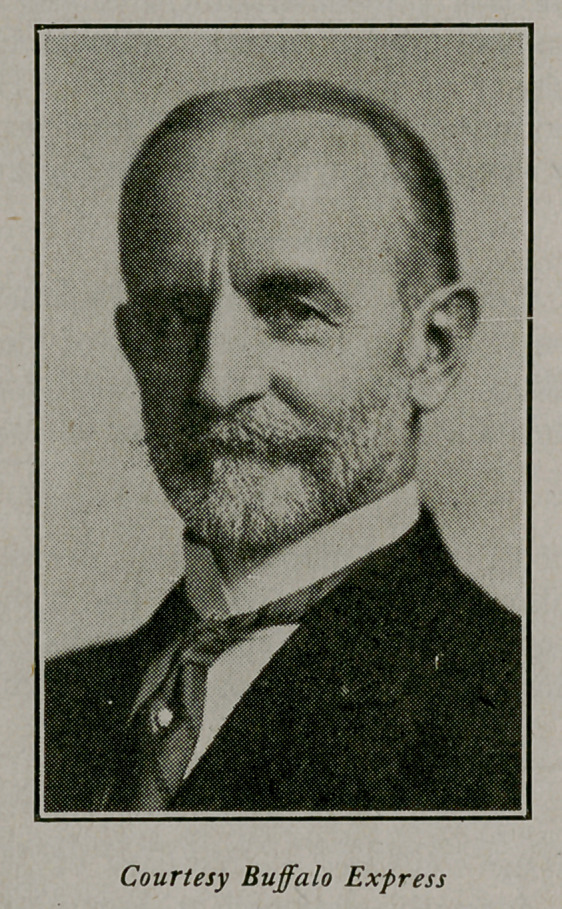# George Raynolds Stearns

**Published:** 1913-09

**Authors:** 


George Raynolds Stearns was born in Buffalo, March 20, 1853. He was graduated with honors from the Central High School in 1871 and as A. B. from the University of Rochester in 1875, being elected to the Phi Beta Kappa. He secured the degree of M. D. from the New York Homeopathic College in 1878 and was granted the honorary degree of A. M. by Rochester the same year. After a year's service at Ward's Island Hospital he returned to Buffalo, where he has since been in practice until his untimely and tragic death, August 8, due to being struck by a trolley car. Dr. Stearns was a charter member of the University Club, a member of the Buffalo Society, of the Sons of the Revolution, and, for twenty years an elder in Lafayette Presbyterian

*Courtesy Buffalo Express*

Church. He was a member of the various local, state and national Homoeopathic organizations and also of the Buffalo Academy of Medicine. He took an active part in the establishment of the Homeopathic Hospital and was, at the time of his death, president of the training school. He was formerly a city physician and a member of the staff of Ingleside Home. Dr. Stearns was a good example of the fallacy that a physician cannot secure the highest honor in his native community. He was an able and, in the best sense, a successful physician, a man of scholarly tastes and broad professional views which won him esteem, respect and fellowship among physicians, irrespective of school practice. Among the various marks of this esteem was the presidency of the Western N. Y. Homeopathic Medical Society. He was a gentleman to the core, courteous, affable and interesting as a companion, beloved by his patients, family and intimate friends, held in affectionate regard by a great many whose acquaintance was brief and casual.

## Figures and Tables

**Figure f1:**